# Reconstruction of a replication-competent ancestral murine endogenous retrovirus-L

**DOI:** 10.1186/s12977-018-0416-3

**Published:** 2018-05-02

**Authors:** Daniel Blanco-Melo, Robert J. Gifford, Paul D. Bieniasz

**Affiliations:** 1 Laboratory of Retrovirology and Howard Hughes Medical Institute, The Rockefeller University, New York, NY USA; 2 MRC-University of Glasgow Centre for Virus Research, Glasgow, UK; 3 Present Address: Department of Microbiology, Icahn School of Medicine at Mount Sinai, New York, NY USA

## Abstract

**Background:**

About 10% of the mouse genome is composed of endogenous retroviruses (ERVs) that represent a molecular fossil record of past retroviral infections. One such retrovirus, murine ERV-L (MuERV-L) is an *env*-deficient ERV that has undergone episodic proliferation, with the most recent amplification occurring ~ 2 million years ago. MuERV-L related sequences have been co-opted by mice for antiretroviral defense, and possibly as promoters for some genes that regulate totipotency in early mouse embryos. However, MuERV-L sequences present in modern mouse genomes have not been observed to replicate.

**Results:**

Here, we describe the reconstruction of an ancestral MuERV-L (ancML) sequence through paleovirological analyses of MuERV-L elements in the modern mouse genome. The resulting MuERV-L (ancML) sequence was synthesized and a reporter gene embedded. The reconstructed MuERV-L (ancML) could replicate in a manner that is dependent on reverse transcription and generated de novo integrants. Notably, MuERV-L (ancML) exhibited a narrow host range. Interferon-α could reduce MuERV-L (ancML) replication, suggesting the existence of interferon-inducible genes that could inhibit MuERV-L replication. While mouse APOBEC3 was able to restrict the replication of MuERV-L (ancML), inspection of endogenous MuERV-L sequences suggested that the impact of APOBEC3 mediated hypermutation on MuERV-L has been minimal.

**Conclusion:**

The reconstruction of an ancestral MuERV-L sequence highlights the potential for the retroviral fossil record to illuminate ancient events and enable studies of the impact of retroviral elements on animal evolution.

**Electronic supplementary material:**

The online version of this article (10.1186/s12977-018-0416-3) contains supplementary material, which is available to authorized users.

## Background

Uniquely among animal viruses, retroviruses integrate into the genome of the host cell as an obligate step in their replication cycle. Because the target cells of some retroviruses can include cells of the germ line, proviruses can occasionally become vertically inherited [1]. A subset of these inherited proviruses can become fixed in the population through genetic drift, or sometimes by providing an evolutionary advantage to the host. Inherited proviruses are termed endogenous retroviruses (ERVs) and are present in all animal species that have been examined, accounting for approximately 8 and 10% of the human and mouse genomes, respectively [2]. In nearly every case, however, fixed proviruses have inactivating mutations that prevent their further spread.

The vast array of ERVs represent an extensive viral fossil record that provides an opportunity to study the biology of ancient or extinct retroviruses, and the effects that these viruses have had on the evolution of their hosts [3]. Previously, we and others have reconstructed a full-length infectious human ERV (HERV-K) [4, 5], functional capsid proteins of endogenous chimpanzee gammaretroviruses (CERV 1 and 2) [6, 7], and lentiviruses, PSIV and RELIK [8], as well as functional envelope proteins from CERV2 and HERV-T [9, 10]. These reconstruction experiments have enabled the identification of ancient virus receptors [9, 10] and demonstrated the ancient origin of cyclophilin A-lentiviral capsid interactions [8]. Additionally, these studies have shown that the replication of HERV-K, CERV1 and CERV2 was affected by the APOBEC3 cytidine deaminases [5, 6, 11]. Overall these “paleovirological” studies have provided previously inaccessible insights into the co-evolution of viruses and hosts.

**Fig. 1:**
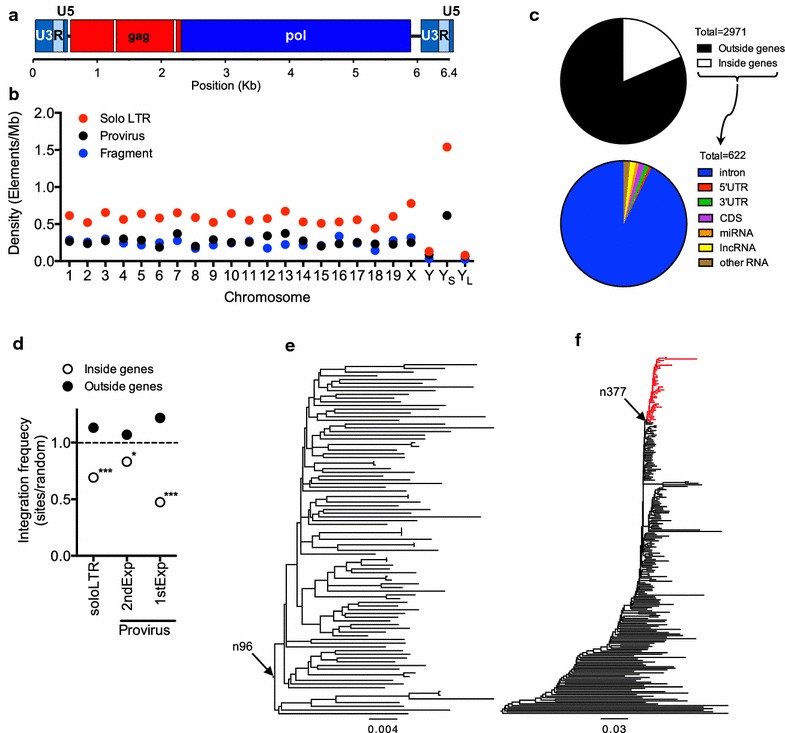
Structure and distribution of MuERV-L elements in the mouse genome and reconstruction of an ancestral ~ 2MY old MuERV-L sequence. **a** Schematic representation of the structure of MuERV-L elements. White boxes in *gag* represent the 33 and 39nt deletions in *gag* at nucleotide positions 671 and 1597, respectively. **b** Distribution of distinct MuERV-L structures among mouse chromosomes. Y_S_ = Y chromosome short arm, Y_L_ = Y chromosome long arm. **c** Distribution of MuERV-L elements in mouse genomic features (GRCm38/mm10). The fraction of the 2971 elements inside and outside genes is depicted (top chart), as the fraction of elements present in each type of gene or gene feature (bottom chart). **d** Distribution of MuERV-L elements in genic or intergenic regions relative to random controls. The measured value indicates the percentage of MuERV-L elements in each population divided by that of the random controls (see “Methods”). The horizontal dotted line indicates no difference between the ratio of MuERV-L elements in each population and that of the controls. (*) *p* value < 0.05. (***) *p* value < 0.001. *P* values are based on Chi-squared goodness-of-fit or contingency table tests. **e** Maximum likelihood phylogenetic tree of 95 LTR–*gag*–*pol*–LTR MuERV-L elements in the mouse genome. Arrow denotes the ancestral node reconstructed by baseml (*pol* and LTR, node 96). **f** Maximum likelihood phylogenetic tree of 230 gag–pol containing MuERV-L elements in the mouse genome. The monophyletic red clade contains only elements with a 33nt deletion in *gag* at position 671 with or without an additional 39nt deletion in *gag* at position 1597. Arrow denotes the ancestral node reconstructed by baseml (*gag*, node 377)

Murine ERV-L (MuERV-L) is an abundant mouse ERV that is transcriptionally active at an early (2-cell) stage of the mouse embryo [12–15]. Previous analyses have established that MuERV-L underwent two amplification bursts, one after the divergence of the *Mus* and *Rattus* genera around ~ 10 million years ago (MYA), and a more recent and prolific burst about 2 MYA, which is distinguished by the presence of a 33nt in-frame deletion in the 5′ half of the *gag* ORF (Fig. 1a) [16]. These amplifications led to the deposition of thousands of MuERV-L derived sequences in the mouse genome. Moreover, MuERV-L belongs to a larger family of ERV-L elements that have been active throughout the evolution of mammals [17–19]. In contrast to their human counterparts, many MuERV-L elements have complete coding potential, encoding open reading frames (ORFs) for *gag* and *pol* (Fig. 1a) [20]. However, like other ERV-L elements, MuERV-L is characterized by the complete absence of an *env* gene that, coupled with its highly restricted early transcription profile, suggests an entirely intracellular retrotransposon-like replication cycle [21]. Indeed, MuERV-L transcripts are able to give rise to intracellular viral-like particles that accumulate in the endoplasmic reticulum [13] but are not thought to be replication competent.

MuERV-L related or derived sequences appear to have been co-opted for two distinct biological activities in the mouse. The antiretroviral restriction factor Fv1, which inhibits infection by MuLV and certain other retroviruses is derived from MuERV-L-like Gag sequences and appeared in the mouse genome at least 5 MYA [22–24]. Additionally, recent studies have suggested that the propensity of MuERV-L to be transcriptionally active only at the two-cell stage of mouse embryogenesis may have led to the co-option of its long terminal repeats (LTRs), as promoters of genes involved in the zygotic genome activation [14, 15]. Transcriptional activity of MuERV-L LTRs in two-cell mouse embryos may drive the expression of hundreds of genes that contribute to the totipotency of the blastomeres, but also results in the expression of MuERV-L Gag–Pol polyprotein and the formation of intracellular viral-like particles [13–15]. As development progresses, MuERV-L LTRs appear to be silenced [14, 25, 26]. The expression of MuERV-L at the two-cell stage does not induce an increase in their copy numbers, suggesting that the expressed proviruses do not have the potential to re-integrate into the genome [25].

In fact, no extant copy of MuERV-L has been demonstrated to be capable of completing a replication cycle. Therefore, we set out to derive a replication-competent MuERV-L, based on the premise that an ancestral reconstruction would deliver a sequence that most closely resembles that of a functional ancestor. Herein, we describe the analysis of MuERV-L elements in the mouse genome, a successful reconstruction of a ~ 2MY old replication-competent ancestral sequence and an analysis of its replication and its interaction with the components of the host intrinsic/innate immune systems.

## Methods

### Bioinformatic analyses and ancestral reconstruction

Screening for MuERV-L elements was performed using amino acid and nucleotide sequences from the MuERV-L reference sequence (MuERV-L^ref^, GenBank: Y12713) [20] as probes for tBLASTn (*gag* and *pol*) and BLASTn (LTR) [27] searches of two mouse genome assemblies: Mm_Celera (NCBI: GCF_000002165.2) [28] and GRCm38/mm10 (UCSC: mm10) [29]. To avoid the identification of sequences from related but distinct retroviruses, BLAST hits with an e-value ≤ 1e−10 were used as probes for a second round of BLASTx or BLASTn searches against a previously constructed database of endogenous and exogenous class III retroviral sequences. Results from these BLAST searches were imported into a relational database to facilitate the management of the screening process and analysis of hits. Reciprocal hits to MuERV-L^ref^ were first ordered by chromosome and orientation and then adjacent or overlapping hits were assembled into proviral loci by comparison with the MuERV-L^ref^ sequence, allowing for insertions no longer than 10,000 nucleotides. The resulting MuERV-L loci were annotated as genomic features of GRCm38 (downloaded from Ensembl [30] using BioMart) by comparison of their chromosome location using in-house Perl scripts.

Dates of integration for MuERV-L elements were estimated by determining the divergence (K) to a consensus sequence (for solo LTRs) or between paired LTRs (for provirus-containing loci) using PAUP* [31], divided by 2× the mouse neutral substitution rate (r) of 4.5 × 10^−9^ substitutions per site per year [29] (K/2r) [32, 33]. The mean sequence identity between the consensus LTR sequence and each of the solo LTRs was 95.43%, with very few outliers (1.5% of solo LTRs showed less than 80% identity), suggesting that the consensus sequence is adequate to perform these estimations. Nevertheless, the estimated ages derived using this method represent approximations to the integration dates and should be treated as such.

Statistical analyses of the positions of MuERV-L elements relative to mouse genomic features were performed using the Pearson’s Chi-squared test for count data (chisq.test) implemented in R. As controls, two sets of random coordinates in the mouse genome were computationally generated using in-house Perl scripts and the runif function implemented in R. For solo LTR comparisons, 10,000 random mouse sequences of 500 nucleotides in length were generated. For proviral loci comparisons, 5000 random mouse sequences of 6500 nucleotides in length were generated. The coordinates of both MuERV-L integrations and random sequences were then mapped to the GRCm38 genome assembly to determine overlapping genomic features (intergenic regions, genes, common repeats and others) using in-house Perl scripts. For ancML integration comparisons, in-house Perl scripts were used to generate controls, consisting of 1000 randomly selected EcoRV-containing fragments (of 1000 nucleotides in length) from the Chinese hamster genome. Each ancML integration site was matched with three of these genomic sequences such that control sites were equidistant from an EcoRV site as was each ancML integration site, as described in [34]. These sites were then mapped to the Chinese hamster genome (criGri1) [35] to determine the overlapping genomic features.

Ancestral reconstruction of MuERV-L was performed using two distinct sequence sets. For the reconstruction of the ancestral *pol* gene and the LTRs, we used a set of 95 complete proviral sequences (LTR–*gag*–*pol*–LTR) identified by default-parameter based BLASTn searches of GRCm38 using MuERV-L^ref^. Each proviral sequence was individually aligned to MuERV-L^ref^ using MUSCLE [36] and a multiple sequence alignment (MSA) was generated using the profile alignment function of MUSCLE. Insertions relative to MuERV-L^ref^ were eliminated from the MSA, except a 6nt insertion at position 298 and 6249 in both LTRs that was shared between 25% of the sequences. The MSA was used to construct a maximum likelihood (ML) phylogenetic tree using raxML [37] with the following parameters: rapid bootstrap analysis with 1000 replicates under GTRCAT followed by a ML search under GTRGAMMA to evaluate the final tree topology (-m GTRCAT -# 1000 -x 13 -k -f a). Thereafter, the tree was midpoint rooted. The MSA together with the phylogenetic tree were used to guide an ML ancestral reconstruction using baseml from the PAML package [38] (model: REV, initial values of alpha and kappa were calculated on the MSA by jmodeltest [39], branch lengths were used as initial values). For the ancestral reconstruction of the *gag* ORF we first aligned and constructed a phylogenetic tree using 230 *gag*–*pol* containing sequences (identified by our screening of the mouse genome described above). We determined the presence or absence of the 33 and 39nt deletions in the *gag* ORF relative to MuERV-L^ref^ (that does not show any deletion) and identified a monophyletic clade of 40 sequences that had the 33nt deletion in *gag* (irrespective of the status of the 39nt deletion). Thereafter, reconstruction of the ancestral *gag* sequence corresponding to the internal node for this monophyletic clade was performed as described above. A correction for the effect of methylation-induced mutations at CpG dinucleotides was applied on both strands of all three sequences (*gag, pol* and LTR) as described in [8]. Specifically, if a particular site where the ancestral reconstruction estimated a TG dinucleotide but at least 10% of the sequences in the MSA encoded a CG at that position, the TG state was considered to be the result of methylation-induced mutation and the sequence at this position was assigned as CG. The resulting sequences were combined to produce the sequence from which ancML was derived.

Hypermutation analysis and statistics were performed using Hypermut 2.0 [40] on the set of 230 gag–pol containing sequences used to reconstruct an ancestral *gag,* using either default parameters or with exclusion of sites with a 5′ C next to the mutated G.

### Plasmid construction

To construct ancML, sequences from the U3 region of the MuERV-L 5′LTR 5′ to the TATA box were substituted with corresponding CMV promoter sequences. We also added an extra 12nt containing two MluI sites immediately 3′ to the *pol* stop codon to facilitate the insertion of a reporter gene. The modified ancML sequence was synthesized and inserted into pUC57 (Genewiz, NJ). The replication dependent LINE-1 element (L1.3 plasmid) [41] was kindly provided by Dr. John V. Moran. The replication dependent *neo* cassette (a *neo* gene controlled by a SV40 promoter and interrupted by an intron) was amplified by PCR from the L1.3 plasmid and inserted into ancML using the MluI sites at the 3′ end of *pol*. A separate pCR3.1 based plasmid, expressing GFP (from a CMV promoter) and a *neo* gene (NEO, expressed from a SV40 promoter) was used as a control.

The ancML-RTmut construct was created by using overlapping PCR and primers that annealed to the RT active site with four nucleotide mismatches, the PCR fragment was inserted into ancML using unique surrounding BstZ17I and NheI restriction sites contained in the outmost primers, generating an ancML with a mutated RT active site (YIDD to AIAA).

The ancML∆GAAGT construct was generated using PCR and a reverse primer that annealed to the 5′ end of the PBS and the 3′ end of the U5 region of the 5′LTR, and lacked the intervening 5nt linker sequence (GAAGT). The PCR fragment was inserted into ancML using unique AgeI and KpnI restriction sites contained in the forward and reverse primers, respectively.

A plasmid expressing mouse APOBEC3 (mA3, C57BL/6J strain) was kindly provided by Rachel Liberatore (unpublished). A C-termini HA-tagged version of mA3 was produced by PCR using primers containing two HA tags and a 15nt linker sequence, following previously published functional human HA-tagged APOBEC3 proteins [42, 43]. This construct was introduced into the retroviral expression plasmid LBCX using unique SfiI sites. A retroviral vector (LBCX) expressing Fv1^bbn^ was kindly provided by Dr. Theodora Hatziioannou [44].

### Cell culture

Cell lines (except CHO-K1 and pgsA cells) were maintained in Dulbecco’s Modified Eagle Medium (DMEM), Eagle’s Minimum Essential Medium (EMEM) or Roswell Park Memorial Institute medium (RPMI) supplemented with 10% FBS and gentamycin (2 µg/ml, Gibco) according to ATCC instructions. CHO-K1 and pgsA cells were maintained in Ham’s F-12 media supplemented with 10% FBS, 1 mM of l-glutamine and 2 µg/ml of gentamycin. All cells were incubated at 37 °C, except DF-1 cells that were incubated at 39 °C.

### Generation of CHO cell lines expressing murine APOBEC3

293T cells were transfected (using polyethylenimine) with plasmids expressing MuLV gag–pol, and VSV-G, along with an LBCX based retroviral vector expressing HA-tagged mA3 or Fv1^bbn^. Viral stocks were harvested and filtered (0.22 μm) 2 days after transfection, and were used to transduce CHO-K1 cells (seeded in 24 well plates). Transduced cells were expanded in 10 cm dishes with media supplemented with 5 μg/ml of blasticidin (Thermo Fisher Scientific Inc.). Single cell clones expressing mA3 were isolated by seeding blasticidin resistant cells at 0.5 cells per well in a 96 well plate. Three distinct single clones that expressed mA3 in 100% of the cells (tested by immunofluorescence) were used in ancML replication assays.

### Immunofluorescence assay

Individual clones of CHO cells expressing murine APOBEC3 were fixed with 4% paraformaldehyde (PFA) for 30 min followed by treatment with 10 mM glycine (diluted in PBS) for another 30 min. Cells were permeabilized with a buffer containing 0.1% of Triton X-100 and 5% goat serum (diluted in PBS) for 15 min. Cells were then washed 2 times with PBS before being treated with mouse monoclonal anti-HA antibody (Covance) diluted in a buffer containing 0.1% Tween-20 and 5% goat serum (diluted in PBS) for 2 h at room temperature. Cells were washed three times with PBS before being treated with goat anti-mouse secondary antibody (Alexa Fluor 488 dye, ThermoFisher) diluted in a buffer containing 0.1% Tween-20 and 5% goat serum (diluted in PBS) for 1 h at room temperature. Cells were washed three more times with PBS and fluorescent microscopy images were analyzed using the EVOS FL Cell Imaging System.

### MuLV infection assay

293T cells were transfected (using polyethylenimine) with plasmids expressing N-tropic or B-tropic MuLV gag–pol, and VSV-G, along with a CNCG based retroviral vector expressing GFP [45]. Viral stocks were harvested 2 days after transfection, filtered (0.22 μm) and were used to infect control or Fv1^bbn^-expressing CHO cells. Two days post infection the percentage of GFP positive population was quantified using the Guava EasyCyte flow cytometer (Millipore).

### MuERV-L(ancML) replication assays

The cell lines listed in Table 2 were seeded in 12 well plates 1 day before being transfected with 700 ng of plasmids containing L1.3, ancML or a plasmid expressing *gfp* and a *neo* gene, using 4 μl of Lipofectamine 2000 (Thermo Fisher Scientific Inc.) according to manufacturer instructions. Two days after transfection, cells were plated in 6-well plates with G418 selection media (containing concentrations of G418 that were previously calibrated for each cell type). Ten days later, surviving cells were fixed with 4% PFA and colonies were stained using 0.3% crystal violet in 20% ethanol for counting.

Subsequently, the ancML replication assays were routinely done using CHO-K1 cells as follows. CHO-K1 cells were seeded at 3 × 10^5^ cells per well in a 12 well plate. One day later the cells were transfected with 1 μg of plasmid DNA, using 3 μl of Transit-CHO supplemented with 0.5 μl of CHO-mojo reagent (Mirus) diluted in Opti-MEM (Gibco). One day later, the cells were expanded on a 10 cm dish with media supplemented with or without AZT (obtained through the NIH AIDS Reagent Program, Division of AIDS, NIAID, NIH) or mouse IFNα (Pestka Biomedical Laboratories, Inc.). Two days later, cells were plated in a 15 cm dish or three 96 well plates (for analysis of single cell clones) with media supplemented with 1 μg/ml of G418. For controls, 1/1000 of the cells transfected with the NEO plasmid were plated in the 15 cm dish with selection media. Cells in 15 cm dishes were cultured under selection for 10 days before treatment with 4% PFA and colonies were stained with 0.3% crystal violet in 20% ethanol for counting. Single colonies in 96 well plates were monitored and expanded until reaching confluence in a 10 cm dish. Genomic DNA (gDNA) was extracted from 5 × 10^6^ cells using QIAmp DNA mini kit (QIAGEN) for analysis of ancML integration (see below).

To determine the fate of the intron interrupting the *neo* gene during ancML replication, gDNA extracted from CHO pools of cells transfected with a plasmid expressing ancML, ancML∆GAAGT or an empty vector, was used as template for PCR analysis. Forward and reverse primers were design to anneal to the extreme 5′ and 3′ ends of the *neo* gene. For all PCRs performed in this study we used Phusion High-Fidelity DNA Polymerase (Thermo Fisher Scientific Inc.).

### Integration site analyses

Sites of ancML integration were determined using a universal Genome Walker kit (Clontech). Briefly, gDNA was extracted from expanded single cell clones of CHO cells following transfection with a plasmid containing ancML and selected in G418. The gDNA was digested with EcoRV (New England Biolabs) and ligated to adaptors. Nested PCRs were performed using forward primers that were designed to anneal to the R region of the 3′LTR and the reverse primers to the adaptor sequence, thereby amplifying 3′ flanking sequences. Bands from second round PCR reactions were gel purified and inserted into pCR-Blunt II-TOPO using the Zero Blunt TOPO PCR cloning Kit (Life technologies) for sequencing. To amplify and sequence the 5′ flanking site we use reverse primers specific to the 5′ LTR and designed forward primers that would specifically anneal to the predicted integration site, based on the previously sequenced 3′ flanking sequence. The resulting CHO gDNA sequences were mapped to the CHO genome (criGri1) using BLAT [46] searches on the UCSC genome browser [47]. To account for biases due to location and density of EcoRV restriction sites, we compared the distribution of the 26 ancML integration sites to matched random controls consisting of three random genomic locations that were at the same distance from an EcoRV site as the site found in the flanking CHO DNA sequence for each MuERV-L integration site [34] (see above).

## Results

### Bioinformatic screens for MuERV-L elements in the mouse genome

To construct a replication-competent MuERV-L sequence we first catalogued the diversity of MuERV-L related sequences in the mouse genome. Currently, there are two available complete mouse genome assemblies: the Mouse Genome Reference Consortium build 38 (GRCm38 also known as mm10) corresponding to the C57BL/6J strain [29], and the whole genome shotgun (WGS) assembly (Celera) that corresponds to a mixture of 5 strains (129X1/SvJ, 129S1/SvImJ, DBA/2J, A/J and C57BL/6J) [28]. We mined both genome assemblies using BLASTn and tBLASTn [27] searches with separate *gag*, *pol* and LTR probes from a MuERV-L reference sequence (GenBank: Y12713) [20]. The resulting hits were defragmented by merging contiguous hits that mapped to the same locus, representing individual elements.

Overall, we found nearly 3000 MuERV-L elements in the mouse genome that had three major types of structures (Table 1, Fig. 1b, Additional file 1: Table S1). One type comprised complete or near complete proviruses, consisting of an internal *gag*–*pol* region flanked by two LTRs. The frequency with which this structure occurred was highly discrepant in the two genome assemblies, with 220 proviruses being present in the Celera assembly and 719 in GRCm38 (Table 1). It is unclear whether this discrepancy is due to the different mouse strains or the assembly methods used in each genome project. A second type of MuERV-L elements were solo LTRs that typically arise from recombination between the LTRs flanking a provirus, resulting in the complete excision of the internal sequence. This type of element represents the single most abundant ERV type in animal genomes and accounts for > 50% of the MuERV-L elements in the mouse genome (Table 1). The third type of MuERV-L structures, representing ~ 25% of all elements, were composed of internal sequences with or without a single associated LTR (Table 1).

**Table 1:** MuERV-L sequences identified in mouse genome assemblies

Assembly	Strain	References	Total	soloLTR	Provirus	Fragments
GRCm38	C57BL/6J	[29]	2971	1588	719	664
Mm_Celera	Mixture	[28]	2768	1775	220	773

Because the GRCm38 assembly was better supported by external and internal annotations we utilized this data source to analyze the distribution of MuERV-L elements in the genome (Fig. 1b–d). MuERV-L elements were roughly evenly distributed across mouse chromosomes (Fig. 1b), with the exception of the Y chromosome in which MuERV-L was underrepresented. Indeed, there were only a few elements in the Y chromosome. This finding was surprising, given that other ERVs, (e.g. HERV-K) are enriched in the human Y chromosome [48]. However, in contrast to human Y chromosome, the long arm of the mouse Y chromosome is a highly dynamic gene rich region that has frequently expanded and undergone rearrangement over the past ~ 3 MY [49]. The distribution of MuERV-L elements in this mouse chromosome shows a clear discrepancy between the short and long arms (Fig. 1b). This finding is likely explained by the low recombination rate of the short arm of the mouse Y chromosome, resulting in an enrichment of mobile DNA elements, while recent gene amplification and rearrangement events in the long arm may have inhibited the fixation of MuERV-L elements.

The majority (79%) of MuERV-L elements were found in intergenic regions (Fig. 1c). About ~ 19.5% of elements were found inside introns (Fig. 1c), of which the majority (65%) were found in antisense orientation relative to of the corresponding gene, as previously documented for Intracisternal A particles (IAPs) [50]. The remaining elements (1.5%) were found primarily in non-coding RNA genes and untranslated exons (UTRs, Fig. 1c). Only 10 LTRs were found overlapping coding exons. This distribution differs significantly compared to randomized controls (*p* < 0.001). This enrichment of elements outside genes was also apparent if analysis was confined to solo LTRs (*p* value < 0.001, Fig. 1d) or if proviruses from the 2MYA or 10MYA expansions were analyzed separately (*p* values < 0.05, Fig. 1d). Overall, these observations are consistent with an expected selective pressure against retention of MuERV-L elements in genes, which was less evident for younger loci (2nd expansion, Fig. 1d). Interestingly, 10.13% of proviruses implicated in the 10MYA expansion have lost a recognizable ORF (either *gag* or *pol*), in contrast to integrations implicated in the 2MYA expansion where only 5.02% have lost an ORF, consistent with the notion that a modest selection to purge MuERV-L sequences is ongoing.

### Reconstruction of an ancestral MuERV-L

To reconstruct the ancestral MuERV-L LTRs and the *pol* ORF, we selected 95 complete (LTR–*gag*–*pol*–LTR) proviruses that were most closely related to a reference MuERV-L sequence (defined by BLAST searches), as this sequence has retained coding potential for both ORFs, has almost identical LTRs, and contained recognizable functional motifs [20]. These sequences were used to guide a maximum likelihood (ML) reconstruction of the root node (*pol* n96) and a pair of identical LTRs (Fig. 1e).

**Fig. 2:**
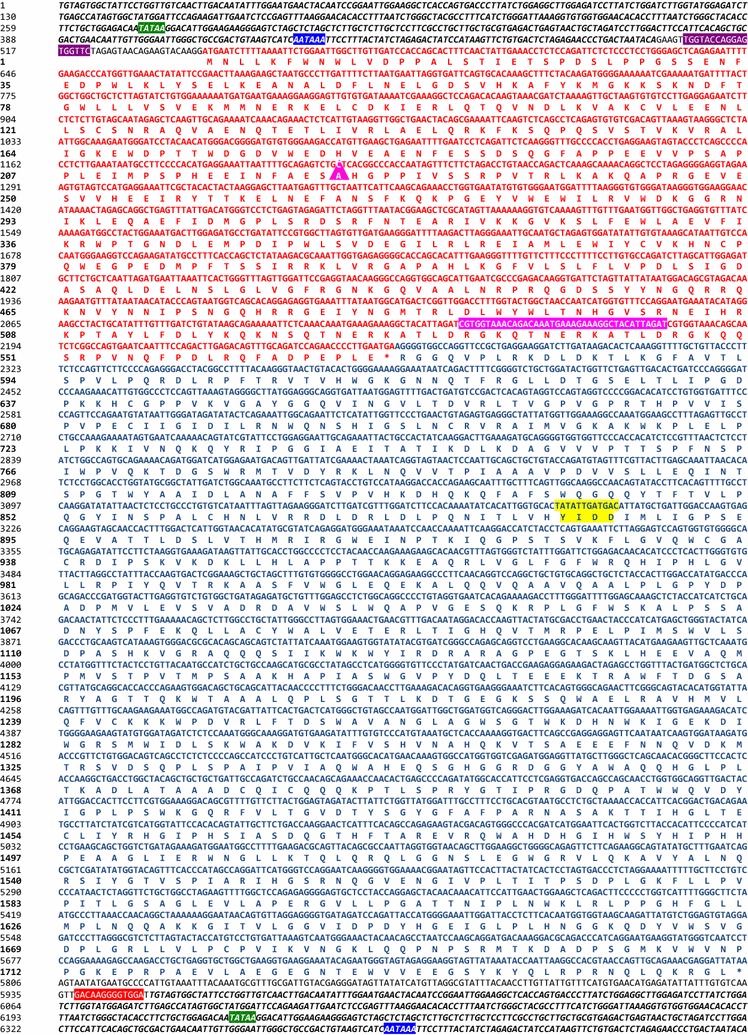
Nucleotide sequence and translation products of a reconstructed ancestral MuERV-L. LTR sequences are shown in bold italics. Nucleotide and protein sequence of *gag* and *pol* are indicated in red and blue, respectively (amino acid single letter code, (*) represents stop codons). The 33nt deletion in gag is shown with a magenta triangle. The position of the 39nt that are deleted in some MuERV-Ls is highlighted in magenta. The RT active site is highlighted in yellow. The PBS is indicated in violet, the polypurine tract in red, the TATA box in green and the polyadenylation site in bright blue

For reconstruction of the Gag gene we took a slightly different approach. As the 2nd expansion was the most prolific and we expected that younger integrations would be less divergent from a functional ancestor than older integrations, we selected *gag* sequences that were specific to the most recent expansion (based on the presence of the 33nt in-frame deletion in *gag*). After aligning 230 *gag*–*pol* containing loci that were present in both Celera and GRCm38 assemblies to the reference sequence (that does not exhibit the deletion), we identified a monophyletic clade of 40 elements that all contained a 33nt in-frame deletion in the 5′ half of their *gag* ORFs (Fig. 1f). The *gag* sequences in this clade were selected to guide a ML reconstruction of this internal node (*gag* n377). Thereafter, the combined ancestral LTR, *gag* and *pol* ancestral sequences were corrected for probable errors derived from deamination of methylated CpG dinucleotides to create a ~ 2 MY ancestral MuERV-L sequence (ancML, Fig. 2 and Additional file 2) (see “Methods”).

### ancML is replication-competent and its replication is dependent on a functional reverse transcriptase

**Fig. 3:**
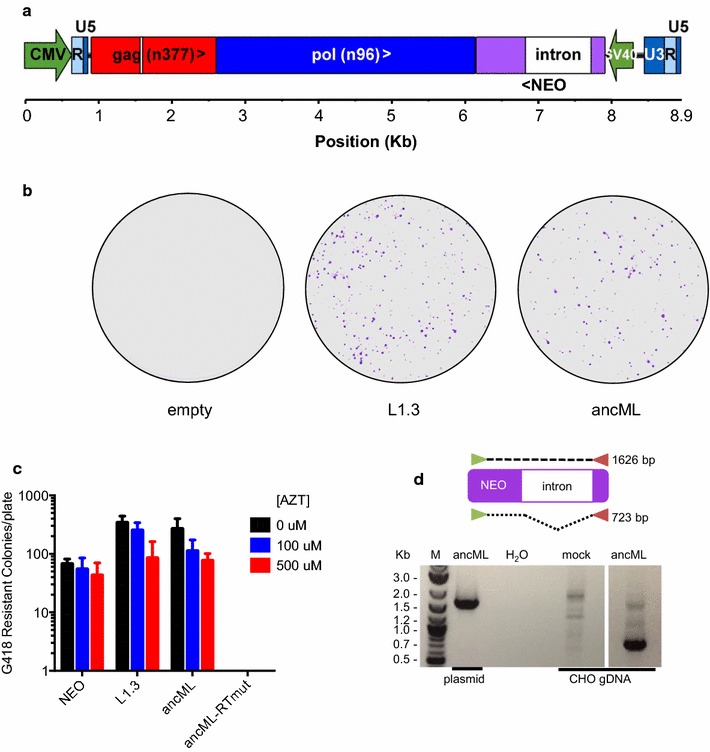
Reverse transcription-dependent ancML replication. **a** Organization of the ancML construct. Green arrows indicate promoter sequences. NEO: *neo* gene in reverse orientation relative to ancML transcription. Chevrons indicate the orientation for each ORF (>: forward, <: reverse). A white box indicates a 33nt deletion in *gag* at position 671. **b** G418 resistant colonies on 15 cm cell culture plates derived from CHO cells transfected with plasmids expressing ancML, L1.3 or an empty vector. **c** Quantification of G418 resistant CHO cell colonies following transfection and treatment in the presence or absence AZT. CHO cells were transfected with plasmids expressing a *neo* gene (NEO), L1.3, ancML or an ancML construct with inactivating mutations in the RT active site (ancML-RTmut). AZT treatment was applied for 2 days before G418 selection. Data are mean ± SD from 3 independent experiments. **d** PCR amplification of the *neo* gene in genomic DNA (gDNA) extracted from CHO cells following transfection with a plasmid expressing ancML or an empty vector. A scheme of the PCR amplification strategy is shown on top. The use of template DNA from plasmid or from CHO gDNA as well as a water control is indicated. M: molecular weight ladder

To assess the potential replication competence of the reconstructed ancestral MuERV-L sequence, we inserted the full-length ancML sequence into a plasmid vector, replacing the U3 region of the 5′ LTR by a CMV promoter to overcome the highly restricted promoter activity of the MuERV-L LTR (Fig. 3a). This design allows for the loss of the CMV promoter sequence after transcription, reverse transcription and integration, resulting in two identical flanking LTRs. A replication-dependent reporter gene, consisting of a *neo* gene controlled by a separate SV40 promoter and interrupted by an intron, was inserted between *pol* and the 3′ LTR (Fig. 3a). While the SV40p-*neo* cassette is in reverse orientation relative to ancML transcription, the splice donor and acceptor sites of the intron are in the same orientation as the ancML transcription. Thus, only if ancML undergoes splicing, reverse transcription and integration in the host cell genome the intron is removed and a functional Neo (G418 resistance) protein is expressed. This approach has been used previously to monitor the intracellular replication of retrotransposons [51].

We determined whether transfection of a plasmid harboring ancML could produce G418 resistant colonies in cultured cell lines. For this purpose we tested a set of 13 cell lines: 6 of primate origin, 5 from rodents, 1 from a carnivore and 1 from an avian species (Table 2). Despite efficient transfection in the majority of the cells tested, and the abundant formation of G418-resistant colonies using a control plasmid, the ancML expression plasmid was able to generate G418 resistant colonies only in Chinese hamster ovary K1 (CHO) cells, CHO-derived pgsA cells, and (to a greatly reduced extent) in Vero cells (Table 2 and Fig. 3b). Surprisingly, a control plasmid expressing a LINE1 element containing the same replication dependent *neo* resistant gene (L1.3 plasmid) [41] failed to produce G418 resistant colonies in 4 of the cells tested, including Vero (Table 2). Conversely, all the cell lines tested generated G418 resistant colonies when transfected with a control plasmid expressing an intact *neo* gene.

**Table 2:** Cell lines tested for replication of ancML

Cell Line	Organism	Lineage	GFP^#^	NEO^a^	L1.3^b^	ancML^c^
DF-1	*Gallus gallus*	Aves	***	+++	++	−
CRFK	*Felis catus*	Carnivora	**	+++	+	−
CV-1	*Cercopithecus aethiops*	Primates	*	++	+	−
Vero	*Cercopithecus aethiops*	Primates	***	+++	−	+
HT1080	*Homo sapiens*	Primates	**	++	+	−
HOS	*Homo sapiens*	Primates	*	++	−	−
Huh7.5	*Homo sapiens*	Primates	*	++	−	−
HeLa	*Homo sapiens*	Primates	***	+++	++	−
pgsA745	*Cricetulus griseus*	Rodentia	**	++	+	++
CHO-K1	*Cricetulus griseus*	Rodentia	**	++	+	++
MusDunni	*Mus dunni*	Rodentia	***	+++	+	−
SC-1	*Mus musculus*	Rodentia	**	++	−	−
NIH3T3	*Mus musculus*	Rodentia	**	++	++	−

All cell lines were transfected using lipofectamine 2000

^#^A plasmid expressing GFP was utilized as a transfection control. Percentage of GFP positive cells: (−) 0%, (*) < 10%, (**) 10–50%, (***) > 50%

^a^A pCR3.1 plasmid expressing a *neo* gene from a SV40 promoter was used as a control for G418 resistant colony formation

^b^The replication dependent LINE1 (L1.3) plasmid [41] was used as a control for retrotransposition and G418 resistance from the interrupted NEO reporter cassette

^c^ancML correspond to a pUC57 plasmid containing the cassette depicted in Fig. 3a

Number of G418 resistant colonies: (−) None, (+) ≤ 10, (++) > 10, (+++) > 50

To determine whether the ancML-associated G418 resistant colonies had arisen as a result of ancML replication, we mutated the predicted ancML reverse transcriptase (RT) active site from YIDD to AIAA (Fig. 2). This mutation completely abolished the production of G148 resistant colonies following ancML transfection (Fig. 3c). Additionally, we found that the formation of G418 resistant colonies by the ancML and L1.3 constructs was modestly reduced in the presence of azidothymidine (AZT), a retroviral RT inhibitor (Fig. 3c), while the formation of G418 resistant colonies by CHO cells transfected with a control plasmid expressing the *neo* gene was nearly unaffected. We isolated genomic DNA (gDNA) from CHO cells that had been transfected with the ancML plasmid and selected in G418, and determined the fate of the intron in DNA forms of ancML by PCR (Fig. 3d). In CHO cells transfected with the ancML plasmid, the vast majority of the amplified DNA sequences corresponded to the properly processed *neo* gene, with the intron excised (Fig. 3d).

Overall, these results indicate that the reconstructed ancestral MuERV-L sequence is replication competent and able to undergo transcription and reverse transcription upon transfection into CHO cells.

### Analysis of ancML integration in CHO cells

**Fig. 4:**
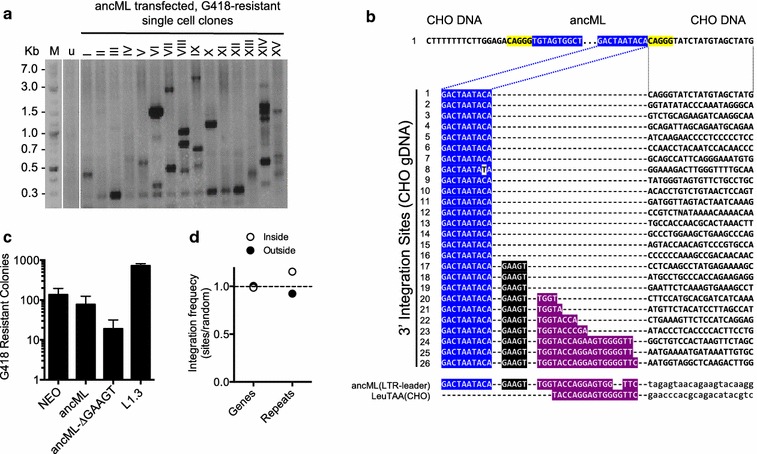
Integration of ancML into CHO cell DNA. **a** Example of a genome walker experiment to determine the 3′ flanking sequence of ancML integration events in 15 single cell clones that became resistant to G418 following transfection with ancML. Nested PCR reactions were done using EcoRV digested, adapter ligated, gDNA from single G418-resistat cell clones. Forward and reverse primers were designed to anneal to the R region of the 3′LTR and to the adaptor sequence, respectively. M: molecular weight ladder. u: CHO DNA without an integrated ancML insertion. **b** Top: the sequence of an integration site with both 5′ and 3′ flanking CHO gDNA. The five-nucleotide target site duplication is indicated in yellow. Bottom: Sequences of 26 ancML integration sites in the CHO genome. Sequences of the ancML U5-PBS region as well as the Leucine (TAA) tRNA sequence are included at the bottom of the diagram. Sequence from the U5 region of the 3′ ancML LTR is indicated in blue. The 5nt linker sequence is indicated in black. The PBS sequence is indicated in purple. CHO genomic sequences are indicted in bold. Dotted lines indicate correspondence of each sequenced 3′ integration junction to the integration site at the top. **c** Enumeration of G418 resistant colonies of CHO cells transfected with plasmids expressing a *neo* gene (NEO), L1.3, ancML and an ancML construct with a deletion of the 5nt linker sequence between the 5′ LTR and the PBS (ancML ∆GAAGT). Data are mean ± SD from 3 independent experiments. **d** Distribution of ancML integration sites in genic, intergenic, or repeat regions relative to matched random controls. The measured value indicates the percentage of ancML integration sites in each population divided by that of the matched random controls (each integration site was matched to three random genomic sequences equidistant to the EcoRV site where the adaptor was ligated). The horizontal dashed line indicates no difference between the frequencies of ancML integration sites in each population compared to the matched controls

We next determined whether ancML underwent bona fide integration into CHO cell DNA. For this purpose we used an adapter ligation-PCR technique (Genome Walker kit, Clontech) to amplify integration sites using primers specific to the MuERV-L LTR and an adaptor sequence. The risk of amplifying CHO genomic DNA from hamster ERV-L LTR sequences was minimal as these LTR sequences are quite divergent from those of ancML (only 55.8% of sequence identity) with indels and substitutions in the annealing sites for the primer sets used. Additionally, hamster ERV-L elements exist only in moderate copy numbers in the Chinese hamster genome [52]. Therefore, DNA from G418-resistant single cell clones of CHO cells, previously transfected with ancML, was digested with restriction enzymes, ligated to linkers and subjected to nested PCR reactions. This procedure revealed bands of varying sizes, sometimes consistent with multiple integrations per cell (Fig. 4a). We cloned and sequenced some of these PCR products. Although some clearly resulted from amplification of the transfected ancML plasmid DNA, we were able to identify 26 *bona*-*fide* integration sites with CHO genomic DNA flanking the 3′ LTR (Fig. 4b and Additional file 3: Table S2). Amplification of sequences flanking the 5′ LTR for one of these integrants revealed a five-nucleotide target site duplication (Fig. 4b), as was observed for MuERV-L sequences present in the mouse genome.

Surprisingly, 10 of the 26 3′ integration sites included a portion of the 5′ leader sequence containing various lengths of the primer-binding site (PBS) and a five-nucleotide LTR-PBS linker sequence, flanking CHO DNA (Fig. 4b). This separation of LTR and PBS is uncommon in exogenous retroviruses and has only previously been observed in another ERV, HERV-E [53]. Elimination of these five nucleotides in the ancML sequence resulted in a ~ 4 fold reduction in the number of G418 resistant colonies, suggesting an enhancing, but non-essential role for the five nucleotide linker in MuERV-L replication (Fig. 4c). Intriguingly, ~ 6.5% of the complete (LTR–*gag*–*pol*–LTR) proviruses in the mouse genome also contain similar sequences (5-nt linker/PBS) at the end of the 3′ LTR, thus showing that this phenomenon also occurred during ancient MuERV-L replication events (Additional file 1: Table S1). During reverse transcription, after the synthesis of the plus-strand strong-stop DNA (+sssDNA), RNase H should remove the primer tRNA, thereby exposing sequences on the +sssDNA that are complementary to the minus strand PBS which will guide the second strand transfer [54, 55]. Inefficient removal of the tRNA primer might result in the synthesis of +sssDNA that includes additional sequences 3′ to the PBS. Such a scenario might explain the unusual integration site structure that we observed for some MuERV-L and ancML insertions (Fig. 4b).

We mapped the position of the 26 ancML integration sites to the Chinese hamster genome using the UCSC genome browser (Fig. 4d) [35, 47]. The Chinese hamster genome (CriGri_1.0) is currently assembled to the scaffold level and has been annotated by distinct de novo, expression-based and homology gene prediction systems [35]. The majority of the ancML integration sites (19/26 sites) corresponded to intergenic regions, 5/26 sites corresponded to introns and one corresponded to exon 3 of *Znf462*. The single remaining site could not be classified as intergenic or in genes because it mapped to multiple scaffolds. Of the 26 integration sites, 10 were in elements corresponding to SINE (4), LINE (4) and ERV-L (2) elements. This distribution of ancML integration sites, i.e. within genes versus intergenic regions, as well as within versus outside repetitive sequences, did not differ significantly from matched randomized controls (*p* value = 0.97 and 0.56 respectively) (Fig. 4d). Although the distribution of the sequenced ancML integration sites and the distribution of MuERV-L elements in the mouse genome appeared different (Figs. 1d and 4b), our ancML integration site dataset was too small to establish statistical significance. Nonetheless, our results suggest that ancML integration sites are random (or close to random) in their distribution in CHO DNA, in contrast to the distribution of MuERV-L proviruses that are found in the mouse genome which have been subject to selection.

### ancML is sensitive to innate host antiviral defenses

**Fig. 5:**
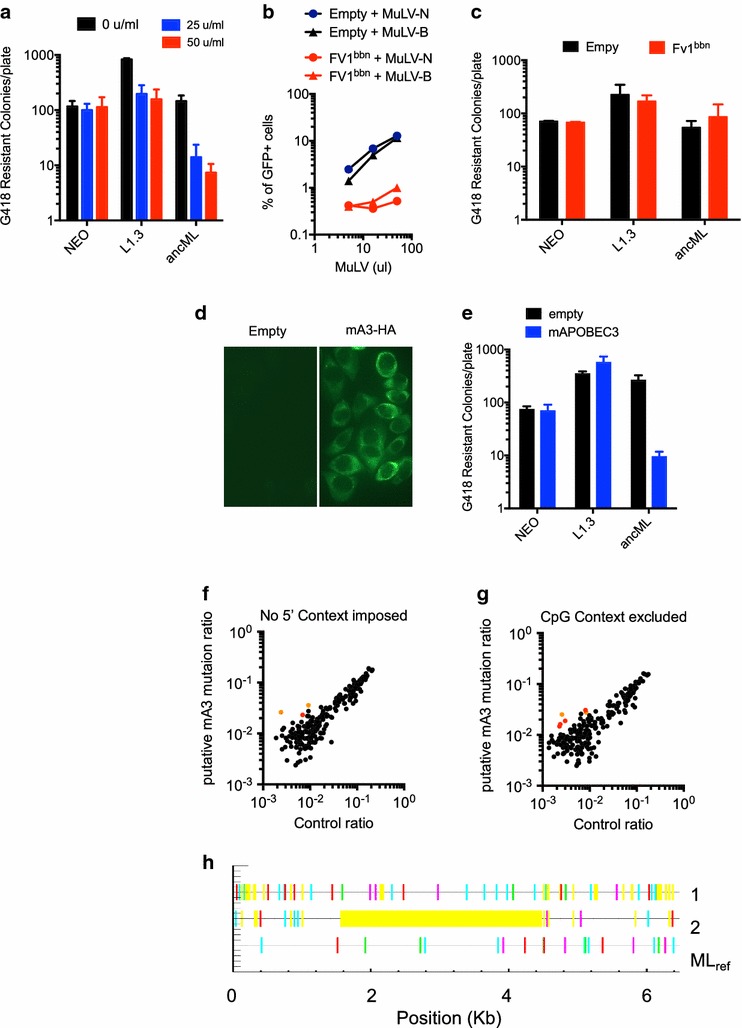
MuERV-L(ancML) replication can be inhibited by innate immune effectors. **a** Enumeration of G418 resistant colonies generated in the presence of increasing amounts of mouse IFNα. CHO cells were transfected with plasmids expressing a *neo* gene (NEO), L1.3, or ancML and cultured with increasing amounts of mouse IFNα for 2 days before G418 selection. Data are mean ± SD from 3 independent experiments. **b** Infectivity of MuLV on CHO cells expressing Fv1^bbn^. Percentage of MuLV infected (GFP positive) cells in CHO cells stably expressing Fv1^bbn^ (red) or an empty vector (black). Circles and triangles indicate infection by N-tropic or B-tropic MuLV, respectively. **c** Enumeration of G418 resistant colonies of CHO cells expressing Fv1^bbn^ or an empty vector were transfected with plasmids expressing a *neo* gene (NEO), L1.3, or ancML. Data are mean ± SD from 2 independent experiments. **d** Representative images of Immunofluorescence assays on CHO cells stably expressing an HA-tagged version of mouse APOBEC3 or an empty vector. CHO cells were fixed with 4% PFA and stained with anti-HA antibodies. **e** Enumeration of G418 resistant colonies of CHO cells expressing mouse APOBEC3. Three clones of CHO cells expressing HA-tagged mA3 or an empty vector were transfected with plasmids expressing a *neo* gene (NEO), L1.3, or ancML. Data are mean ± SD from 3 experiments with independent single cell clones. **f** and **g** Analysis of MuERV-L elements using Hypermut 2.0. Ratio of G to A mutations at preferred mA3 editing sites (RD 3′ to a G) (Y-axis) plotted against ratio of G to A mutations at disfavored mA3 editing sites (YN|RC 3′ to a G) (control ratio, X-axis). No 5′ context was imposed (**f**), or sites with a 5′ C to the mutated G were excluded (**g**). 230 gag–pol containing MuERV-L elements in the mouse genome were compared to their consensus sequence. Data points in red and orange indicate MuERV-L sequences that were statistically significantly enriched in putative mA3 induced mutations (*p* value < 0.05). Data points in orange represent MuERV-L elements that are statistically significantly enriched mA3-induced mutations in both analyses (*p* value < 0.01). **h** Profile of G to A transitions in two putatively mA3-edited MuERV-L proviral sequences compared to a consensus sequence. The profile of the reference MuERV-L sequence is shown for comparison (ML_ref_, non significantly mA3 edited). Lines in red and cyan represent putative mA3-derived G to A transitions, not accounting for the + 2 position (dinucleotide changes from GG to AG and GA to AA respectively), whereas lines in green and magenta represent non mA3-derived G to A transitions (GC to AC and GT to AT respectively). Lines in yellow indicate gaps compared to the consensus sequence

In response to exogenous microbial threats, hosts have evolved sets of genes that sense, and directly interfere with replication of pathogens. One class of such genes which are expressed in response to viral infection following induction by interferons (IFNs), cause a so-called antiviral state [56]. It is not known whether IFNs can inhibit the intracellular replication of retrotransposons. To determine whether ancML replication is affected by type-I IFNs, we transfected CHO cells with plasmids expressing ancML, L1.3 or a *neo* gene and cultured them with media containing varying amounts of murine IFNα (mIFNα) for 2 days prior to selection in G418 (Fig. 5a). The replication of L1.3 was reduced by ~ 4 fold upon mIFN-α treatment, and there was a larger, dose dependent effect on ancML, reaching a ~ 20-fold reduction in G418 resistant colony formation with 50U/ml of mIFNα (Fig. 5a). Notably, generation of G418-resistant colonies by transfected, non-replicated DNA was not affected by mIFNα. Previous studies have observed that mouse IFNα can stimulate an antiviral state in CHO cells [57–60] and promote the induction of hamster ISGs [61]. Thus, these experiments suggest that IFNα is able to inhibit one or more steps in MuERV-L replication. Interestingly pluripotent stem cells have been shown to express a subset of ISGs [62], and suppression of ERV replication may be one impetus for the acquisition of this property.

We also tested whether specific candidate innate immune effectors could inhibit ancML replication. We first tested if the murine restriction factor Fv1 (thought to have been co-opted from a MuERV-L-like element) could have had an impact on MuERV-L replication. For this we constructed CHO cells stably expressing a chimeric form of Fv1 that shows an expanded resistance to different MuLVs (Fv1^bbn^) [63]. As expected, Fv1^bbn^-expressing CHO cells exhibited resistance to infection by both N-tropic and B-tropic MuLV (Fig. 5b). However, Fv1^bbn^-expressing CHO cells supported ancML, or L1.3 replication (Fig. 5c), at levels similar to those of control cells, indicating that ancML is insensitive to this Fv1 protein. We also tested the ability of mouse APOBEC3, that has been previously shown to inhibit endogenous and exogenous retroviruses (reviewed in [64]), to inhibit ancML replication. For this purpose, we generated CHO cell clones that stably expressing the mouse *Apobec3* in 100% of the cells (Fig. 5d). Remarkably, mouse *Apobec3* (mA3) was able to inhibit ancML replication, reducing G418 resistant colony formation by ~ 30-fold, but did not affect L1.3 replication (Fig. 5e). The inability of mA3 to restrict human L1.3 retrotransposition has been previously documented [65], while some human APOBEC3 proteins inhibit L1.3 retrotransposition [42, 66, 67], suggesting that species-dependent differences exist in the ability of APOBEC3 proteins to inhibit the replication of endogenous retroelements.

Because mA3 clearly inhibited ancML replication, and therefore might have affected MuERV-L sequence or replication in vivo, we inspected the 230 gag–pol containing MuERV-L elements that were used to derive ancML *gag* (Fig. 1f) using Hypermut 2.0 [40] (Fig. 5f–h). For each MuERV-L element we compared the number of G to A transitions in mA3-preferred motifs (5′ G(A|G)(A|G|T) 3′) with those in control sites (5′ G(C|T)N 3′ or 5′ G(A|G)C 3′) relative to a consensus sequence (Additional file 4: Table S3). Only three MuERV-L elements showed significant (*p* < 0.05) evidence of mA3 dependent hypermutation when no 5′ context was enforced (Fig. 5f). Because spontaneous deamination of methylated CpG dinucleotides can also produce G to A transitions, we performed the same analysis after excluding sites containing a C nucleotide 5′ to the mutated G. When these sites were excluded, 10 MuERV-L elements showed a significant (*p* < 0.05) evidence for mA3 dependent hypermutation (Fig. 5g). Only two MuERV-L elements exhibited statistically significant evidence of mA3 dependent hypermutation in both analyses (*p* value < 0.01), and both of these elements carried a relatively low mutational burden (Fig. 5f–h). Thus, although ancML replication can be inhibited by mA3, analysis of MuERV-L proviruses in the mouse genome suggests that MuERV-L either rarely encountered mA3, or is inhibited in a manner that prevents the deposition of hypermutated proviruses.

## Discussion

Here, we report the successful reconstruction of a ~ 2MY old replication competent ancestral MuERV-L sequence, through the analysis of a recently expanded subset of fossilized MuERV-L elements in the mouse genome. According to previous studies [16], and corroborated here, MuERV-L originated ~ 10 MYA, after the *Rattus*–*Mus* split and underwent a prolific expansion ~ 2 MYA. In fact, almost 65% of solo LTRs and MuERV-L proviruses identified herein have an estimated integration date of < 3 MYA. Furthermore, the estimated dates of solo LTRs follow a bimodal distribution (a major one centered ~ 3MYA and the other ~ 8MYA) consistent with the estimated times of both expansions (Additional file 1: Table S1). A combination of homology searches and defragmentation methods provided the material for the estimation of the sequence of the ~ 2MY old replication-competent ancestor.

Other highly abundant *env*-defective ERVs typically appear to be derived from closely related elements that possess an *env* gene. While other closely related elements do possess an *env* gene, there are no documented ERV-L elements that encode an *env*. It is likely, therefore, that an ancestral ERV-L element lacked an *env* gene. Thus, the bulk of MuERV-L replication likely occurred through entirely intracellular retrotransposon-like mechanisms [21]. Moreover, the bulk of MuERV-L replication likely occurred in early embryos, as the expression of MuERV-L elements appears to be restricted to the 2-cell embryo, although It is unknown whether this property is confined to the subset of elements that proliferated ~ 2MYA. It is possible that the early embryonic environment is also necessary in some other way for MuERV-L replication given its apparently restricted tropism in cell lines. In particular, it is intriguing that (and as yet unexplained why) MuERV-L only replicated with reasonable efficiency in Chinese hamster ovary cells, even when provided with a promoter that should drive its expression in nearly any cell type.

MuERV-L belongs to an ancient mammalian ERV family (which originated > 100 MYA [18]) that is distantly related to spumaviruses. Therefore, modern functional viral sequences are therefore not useful for attempts to increase the replicative efficiency of ancML. Remarkably, there is a high number of MuERV-L proviruses that have retained their coding potential, and share a high degree of sequence similarity to the functional ancML (with only few coding differences and overall nucleotide identity ranging from 96.16 to 99.31%). However, currently there is no evidence that the ongoing expression of MuERV-L elements at the two-cell stage of the mouse embryo results in successful re-integration, although it is possible that MuERV-L replication and reintegration occurs in modern mouse embryos at some very low rate. Nevertheless, examination of recent *bona fide* integrations might highlight important residues that might be altered to improve ancML replication and/or integration.

We found that mouse IFNα was able to inhibit ancML replication, suggesting that interferon stimulated genes can directly inhibit MuERV-L replication, possibly leading to its recent extinction as a replication competent entity. Alternatively, early embryos may express antiviral proteins that inhibit re-integration of modern MuERV-L elements that would otherwise be intrinsically replication competent [62]. We found that mouse APOBEC3 inhibits ancML replication, but mutational profiles of MuERV-L elements in the mouse genome provide minimal evidence for mA3-dependent hypermutation as a mechanism for inhibition in vivo. During mouse development, mA3 is expressed at the two-cell stage, increasing at the four-cell stage to become one of the top 30% most highly expressed genes [68]. Thus, it is at least possible that mA3 may have acted on replicating MuERV-L elements, perhaps in part through deaminase-independent mechanisms [69].

Despite the apparently random integration pattern of ancML, the analysis of fixed MuERV-L elements showed that there has been a selective pressure to eliminate MuERV-L integrations from genes. Conversely, MuERV-L related sequences (Fv1) have clearly been positively selected to provide defense against retroviral infection [22–24] and recent studies have suggested that regulatory elements of MuERV-L LTRs may have been co-opted to regulate the expression of numerous genes during embryogenesis [14, 15]. While Fv1 arose at least ~ 5–7 MYA, it is unclear whether the potential exaptation of MuERV-L regulatory sequences occurred during the 10MYA expansion or the more recent ~ 2 MYA expansion. Nonetheless, there appears to be both a benefit (co-option for antiviral defense and regulation of embryogenesis) and cost (disruption of gene function) associated with the presence of MuERV-L elements in the mouse genome.

MuERV-L appears to be the only member of the ERV-L family that seems to have been reactivated in recent evolutionary times. It is particularly intriguing that the recent expansion is characterized by an in-frame deletion in *gag*, as it could be this deletion the responsible for releasing some MuERV-L elements from the deleterious effects of a hypothetical inhibitory factor ~ 2MYA. Recent studies have shown the fundamental role that some endogenous retroviral sequences may play in mammalian development and protection from exogenous retroviral infection [15, 23, 24, 70–73]. Indeed one report has suggested that knockdown of MuERV-L transcripts impacts embryonic development [74]. Nevertheless, it remains to be determined whether the current presence of MuERV-L transcripts, proteins and virus-like particles at the two-cell stage of the mouse embryo might be beneficial or deleterious to the mouse.

## Conclusions

The reconstruction of an ancestral MuERV-L sequence highlights the potential for the retroviral fossil record to illuminate ancient events and represents a unique opportunity to study ERV-L biology and reactivation, the role of MuERV-L in mouse development and potentially uncover new roles for ERVs in mammalian biology.

## Additional files

**Additional file 1: Table S1.** MuERV-L loci identified in mouse genome assemblies.

**Additional file 2.**ancML sequence in FASTA format.

**Additional file 3: Table S2.** ancML integration sites cloned from CHO gDNA.

**Additional file 4: Table S3.** Analysis for hypermutation in MuERV-L elements in the mouse genome.
